# The oncogenic potential of a mutant TP53 gene explored in two spontaneous lung cancer mice models

**DOI:** 10.1186/s12885-020-07212-6

**Published:** 2020-08-08

**Authors:** Julian Ramelow, Christopher D. Brooks, Li Gao, Abeer A. Almiman, Terence M. Williams, Miguel A. Villalona-Calero, Wenrui Duan

**Affiliations:** 1 Department of Human & Molecular Genetics, Herbert Wertheim College of Medicine, The Florida International University, Miami, Florida 33199 USA; 2 Biomolecular Sciences Institute, The Florida International University, Miami, Florida 33199 USA; 3 Biological Sciences, College of Arts, Science and Education, The Florida International University, Miami, Florida 33199 USA; 4 Comprehensive Cancer Center at the Ohio State University College of Medicine, Columbus, OH 43210 USA

**Keywords:** Lung cancer, Mouse model, TP53 mutation, Immunotherapy

## Abstract

**Background:**

Lung cancer is the number one cancer killer worldwide. A major drawback in the lung cancer treatment field is the lack of realistic mouse models that replicate the complexity of human malignancy and immune contexture within the tumor microenvironment. Such models are urgently needed. Mutations of the tumor protein p53 are among the most common alterations in human lung cancers.

**Methods:**

Previously, we developed a line of lung cancer mouse model where mutant human TP53-273H is expressed in a lung specific manner in FVB/N background. To investigate whether the human TP53 mutant has a similar oncogenic potential when it is expressed in another strain of mouse, we crossed the FVB/N-SPC-TP53-273H mice to A/J strain and created A/J-SPC-TP53-273H transgenic mice. We then compared lung tumor formation between A/J-SPC-TP53-273H and FVB/N-SPC-TP53-273H.

**Results:**

We found the TP53-273H mutant gene has a similar oncogenic potential in lung tumor formation in both mice strains, although A/J strain mice have been found to be a highly susceptible strain in terms of carcinogen-induced lung cancer. Both transgenic lines survived more than 18 months and developed age related lung adenocarcinomas. With micro CT imaging, we found the FVB-SPC-TP53-273H mice survived more than 8 weeks after initial detection of lung cancer, providing a sufficient window for evaluating new anti-cancer agents.

**Conclusions:**

Oncogenic potential of the most common genetic mutation, TP53-273H, in human lung cancer is unique when it is expressed in different strains of mice. Our mouse models are useful tools for testing novel immune checkpoint inhibitors or other therapeutic strategies in the treatment of lung cancer.

## Background

Lung cancer is considered to be the most common cancer among men when measured on a worldwide basis and has emerged as a leading cause of death among women in more developed countries like the United States [1, 2]. Further, it has been projected that about 228,000 new cases arise and 135,700 deaths will occur in 2020 due to lung cancer [2]. The disease has experienced a huge increase in prevalence in the past decades and is now responsible for approximately 1 out of 5 cancer deaths worldwide, which equates to 19.4% of total cancer deaths [1, 2]. Lung cancer is further categorized into different sub-categories where 75–80% of all lung cancers are non-small cell lung cancers (NSCLC) [3]. It was shown that adenocarcinoma has emerged to be the most common NSCLC subtype [4]. Despite the recent development of many cytotoxic drugs, radiotherapy and patient management, the cure rates for advanced NSCLC remain very low [5, 6]. In fact, there is evidence which suggests that somatic mutations in the genome increases with age, even within stem cells [7–11]. Thus, a comprehensive knowledge of genetic variations that contribute to spontaneous lung cancer development is a necessity for further progress in identifying early interventions and improved clinical treatment.

Around 50–60% of non-small cell lung cancers and 90% of small cell lung tumors contain tumor protein p53 (TP53) mutations, thus TP53 represents one of the most common genetic events in this malignancy [12–14]. Wild type TP53 protein plays a fundamental role in tumor suppression [15, 16] and apoptosis [17]. Upon activation, TP53 can activate specific anti-proliferative responses, including cell-cycle arrest, or apoptosis [17]. Wild TP53 drives these responses primarily by serving as a transcriptional factor that induces gene expression important for each TP53 response. However mutant TP53 have not only lost wild-type TP53 tumor suppressor activity but also gained functions that contribute to malignant progression [14, 17]. The majority of these mutations are missense mutations. Most of the mutations were found within the sequence-specific DNA-binding domain.

Codon 273 of human TP53 is one of the most frequently mutated sites in human lung cancers [18–20]. The human mutant TP53-273H, which has the most common substitution (arginine to histidine), has been shown to have both dominant-negative and gain-of-function properties [21–25]. Unlike most tumor-derived mutant TP53 proteins, TP53-273H retains partial sequence-specific DNA-binding and transcriptional activation functions [26–29]. Thus TP53(273H) could conceivably lead to increased cell proliferation, aberrant DNA recombination, increased genomic instability and reduced chemotherapy efficacy [30–32]. TP53-270H/+ mice (Murine TP53 codon 270 correspond to human TP53 codon 273) developed an increased incidence of carcinomas and B cell lymphomas compared to TP53+/− mice [33]. In addition, this TP53 mutant promotes acceleration of submucosal invasion and metastatic potential of cancer cells in colorectal cancer [34].

To mimic lung cancer development in humans, animal tumor models have been created. The majority are murine models. Previously we have developed a line of transgenic mice where mutant human TP53-273H is expressed in a lung specific manner under the regulation of the alveolar type II cell-specific surfactant protein C (SPC) promoter [35, 36]. Human TP53-273H mRNA and protein were demonstrated specifically in lung tissues. In addition, using the same SPC promoter, we have also created TP53-175H transgenic mice [37]. We have shown that both SPC-TP53-273H and SPC-TP53-175H mice developed lung adenocarcinomas [35–37]. Like human non-small cell lung cancers, formation of the lung adenocarcinomas in these transgenic mice has a latency period and is associated with other gene alterations (e.g. KRAS mutations and p16 gene promoter methylation) [36, 37]. Different from other lung tumor animal models, our model limits the insult to the lung, and mutations in the KRAS gene are acquired mutations, closely mimicking the events that lead to lung cancer development in human patients.

Since both SPC-TP53-273H and SPC-TP53-175H mice are created in an FVB/N strain background, one of the important remaining questions is whether these TP53 mutants have similar oncogenic potential when they are expressed in another strain of mice or more importantly, whether these proposed models can function as treatable models with immune oncologic applications. To answer these questions, we bred the SPC-TP53-273H mice to A/J strain background and created A/J-SPC-TP53-273H transgenic mice. We have also monitored lung tumor development in the FVB/N-SPC-TP53-273H mice with a micro CT to determine the rate of tumor growth. Herein, we report our results.

## Methods

### Generation of transgenic lung cancer mouse model models

**Fig. 1:**
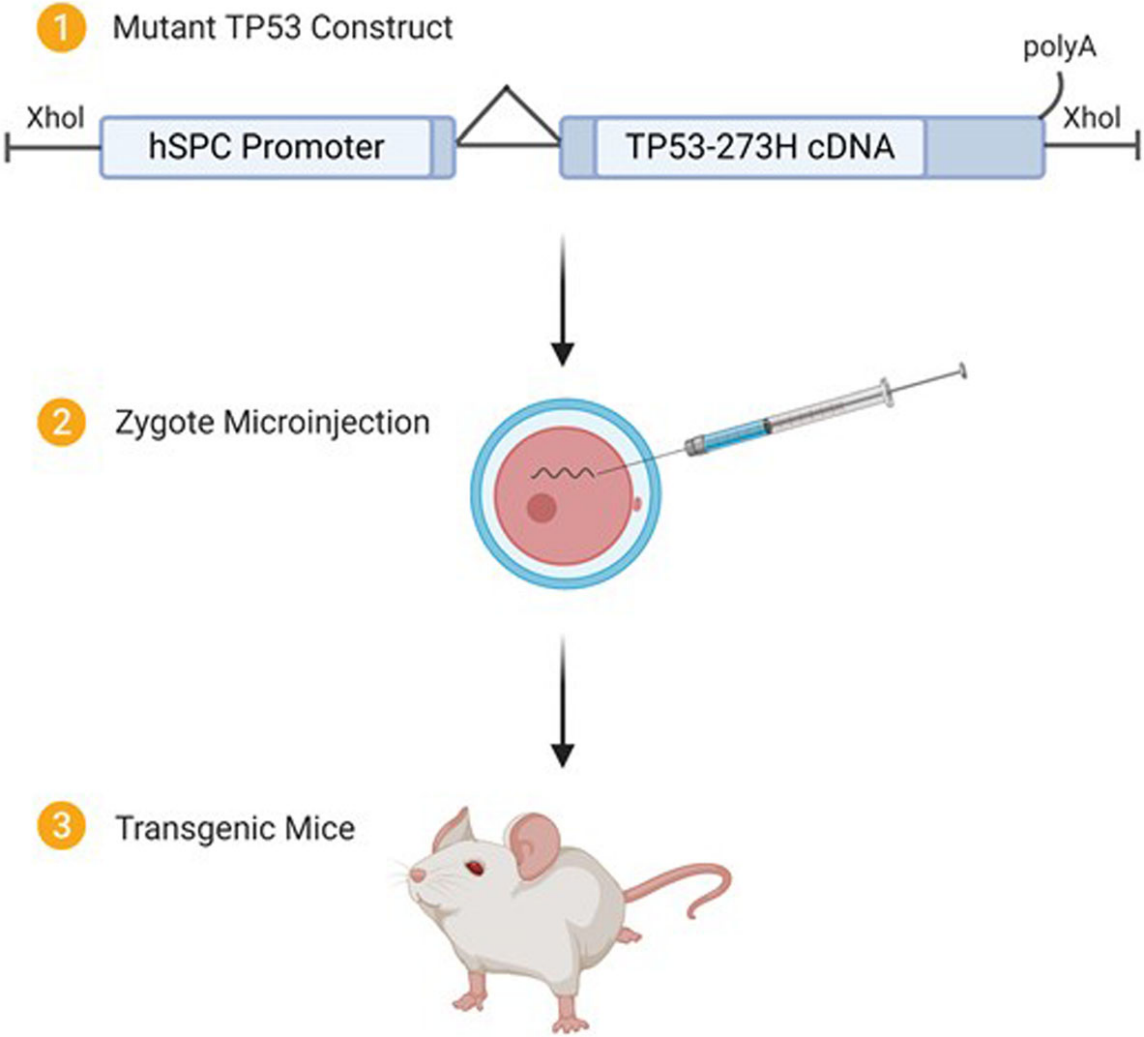
Schematic diagram of creation of the SP-C/p53-273H transgenic mouse. A 1.8-kb human mutant p53-273H cDNA (arginine to histidine substitution at codon 273) was placed under the transcriptional control of a 3.7-kb region of the human SP-C promoter. Transgenic mice were generated by microinjection of a total of 6.7-kb XhoI fragment of the SP-C/p53-273H construct into the pro-nuclei of FVB/N mouse zygotes by standard methods

All animal experiment procedures were conducted in accordance with The Ohio State University Institutional Laboratory Animal Care and Use Committee and the regulations and guidelines of the institutional animal care and use committee (IACUC). Transgenic FVB/N mice were developed using the lung specific human surfactant protein C (SPC) promoter to control expression of mutant TP53(273H) following the standard injection method (Fig. 1) as we described previously [35]. A/J wild-type mice were obtained from Jackson Laboratories (Bar Harbor, ME, USA). The FVB/N-SPC-TP53-273H mice were backcrossed with A/J for 8 generations to obtain A/J-SPC-TP53-273H. Expression of human mutant TP53-273H was confirmed by immunohistochemistry. The presence of the transgene in the subsequent offspring generations were determined by polymerase chain reaction (PCR) using primers as described previously [35].

### Tumors and histopathological assessment

Following dissection, tissue was harvested and inspected for lung tumor and normal tissue. These samples were then processed for histological analysis. Samples were fixed using formalin and embedded in paraffin. Paraffin embedded tissue was sectioned at 4 μm and slides stained with hematoxylin and eosin (H&E). H&E stained sections were evaluated. All tumors observed were processed for histological analysis and 30% of lung samples containing no visible surface tumors were subject to random histological analysis [35].

### Immunohistochemistry analysis

Sections for immunohistochemistry were placed in a 60 °C oven for 1 h, deparaffinized and rehydrated using xylene and graded ethanol solutions, followed by blocking the endogenous peroxidase using 3% hydrogen peroxide. Citric acid solution was used for antigen retrieval at 94 °C using a steamer. Slides were blocked with 10% normal goat serum for 1 h before application of the human TP53-specific DO-7 monoclonal antibody (BD PharMingen, San Diego). The detection system was labeled Streptavidin-Biotin [35]. Slides were observed and images were recorded using an Olympus microscope.

### Lung tumor growth rate estimation

Lung cancer growth rate in mice was evaluated by using the InveonTM system (Siemens, Erlangen, Germany). InveonTM is a 10 cm diameter bore SPECT/PET/CT imaging system. This CT is capable of creating spatial resolutions of < 0.05 mm and is equipped with a real-time reconstruction engine and a post-processing workstation that includes TRI3D-BON (Ratoc System Engineering Co., Ltd., Tokyo, Japan).

The transgenic mice were anesthetized with 1.25% isoflurane and scanned with the micro CT after. The image analysis was done at post processing workstations. Scans were limited to the thorax to optimize resolution and minimize radiation exposure time. Post CT evaluation, the mice recovered for at least 1 h from anesthesia and were then returned to the animal care facility.

### Housing and husbandry

All mice were housed in the University Laboratory Animal Resources (ULAR) facility at The Ohio State University. All experimental procedures were in compliance with the Animal Welfare Act, the NIH Guide for the Care and Use of Laboratory Animals, and other applicable regulations. The animals were cared for by a veterinarian as described in the “Guide for the Care and Use of Laboratory Animals” (NIH Pub. No. 86–23, 1985). Mice were carefully monitored daily. Animals were euthanized with carbon dioxide followed by cervical dislocation. All lungs were examined macroscopically for evidence of tumor formation. A total of 116 A/J and 254 FVB/N mice at the age range of 7–18 months were used for this study.

### Statistical methods

A T-test was used to compare the difference in lung tumor formation between two groups.

## Results

### Increased lung tumor formation in a/J-SPC-TP53-273H transgenic mice

**Fig. 2:**
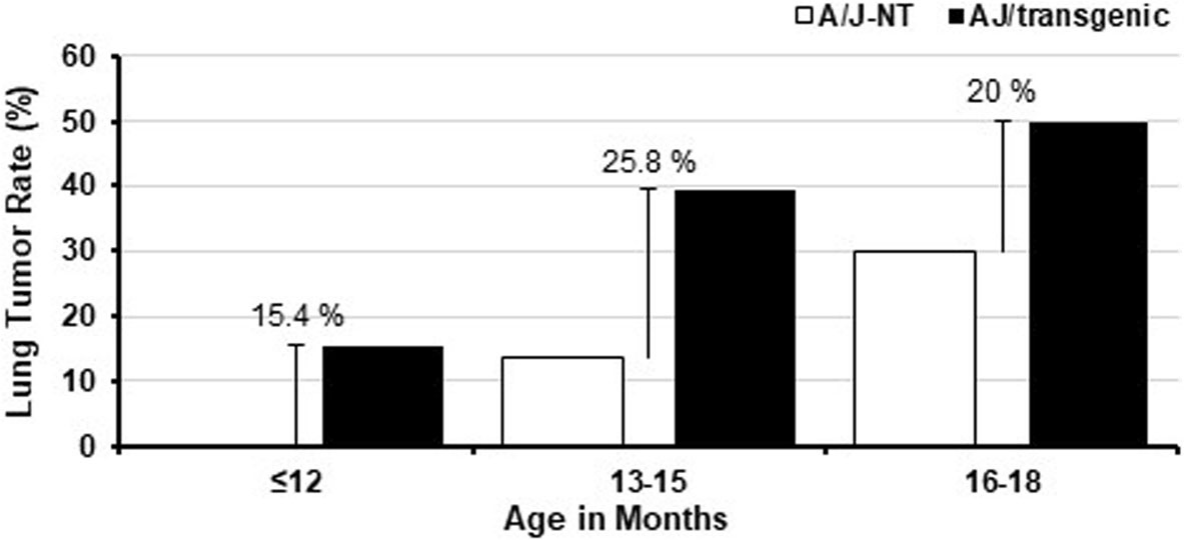
Lung tumor formation in the A/J-SPC-TP53-273H transgenic mice and non-transgenic mice at ages 7–18 months respectively

Generation of the FVB/N-SPC-TP53-273H transgenic mouse was reported previously [35]. Then the FVB/N-SPC-TP53-273H mice were backcrossed with the A/J strain, obtaining A/J-SPC-TP53-273H transgenic mice. Expression of human mutant TP53-273H was confirmed by immunohistochemistry. To evaluate the rate and age of the onset of lung tumors in the A/J-SPC-TP53-273H mice, we evaluated 116 A/J-SPC-TP53-273H mice including 74 transgenic mice and 42 non-transgenic mice by necropsy (Fig. 2).

A total of 25 lung tumors were identified among 74 transgenic mice in the 7–18 months age range. Among the 42 non-transgenic mice, 6 lung tumors were observed. No tumor was observed in both transgenic mice and non-transgenic mice in the 7–9 month cohort. Initial tumor formation was observed between months 10–12 in the transgenic mice; however, lung tumor was not observed until months 13–15 in non-transgenic mice. Tumors rate then increased in months 16–18 in both transgenic and non-transgenic mice (Table 1). Overall, we found that A/J-SPC-TP53-273H transgenic mice had a higher lung tumor rate when compared to their parental strain, the A/J non-transgenic (A/J-NT) mice.

**Table 1:** Lung tumor formation in A/J strain of mice

Tumor	Month ≤ 12	Month 13–15	Month 16–18	***Total***
**A/J-SPC-TP53-273H**				
Yes	2	13	10	25
No	19	20	10	49
**Number of mice**	21	33	20	74
**Tumor rate**	**0.10**	**0.39**	**0.50**	**0.34**
**A/J-NT**				
Yes	0	3	3	6
No	10	19	7	36
**Number of mice**	10	22	10	42
**Tumor rate**	**0.00**	**0.14**	**0.30**	**0.14**

### Comparison of lung tumor formation between FVB/N-SPC-TP53-273H and a/J-SPC-TP53-273H mice

**Fig. 3:**
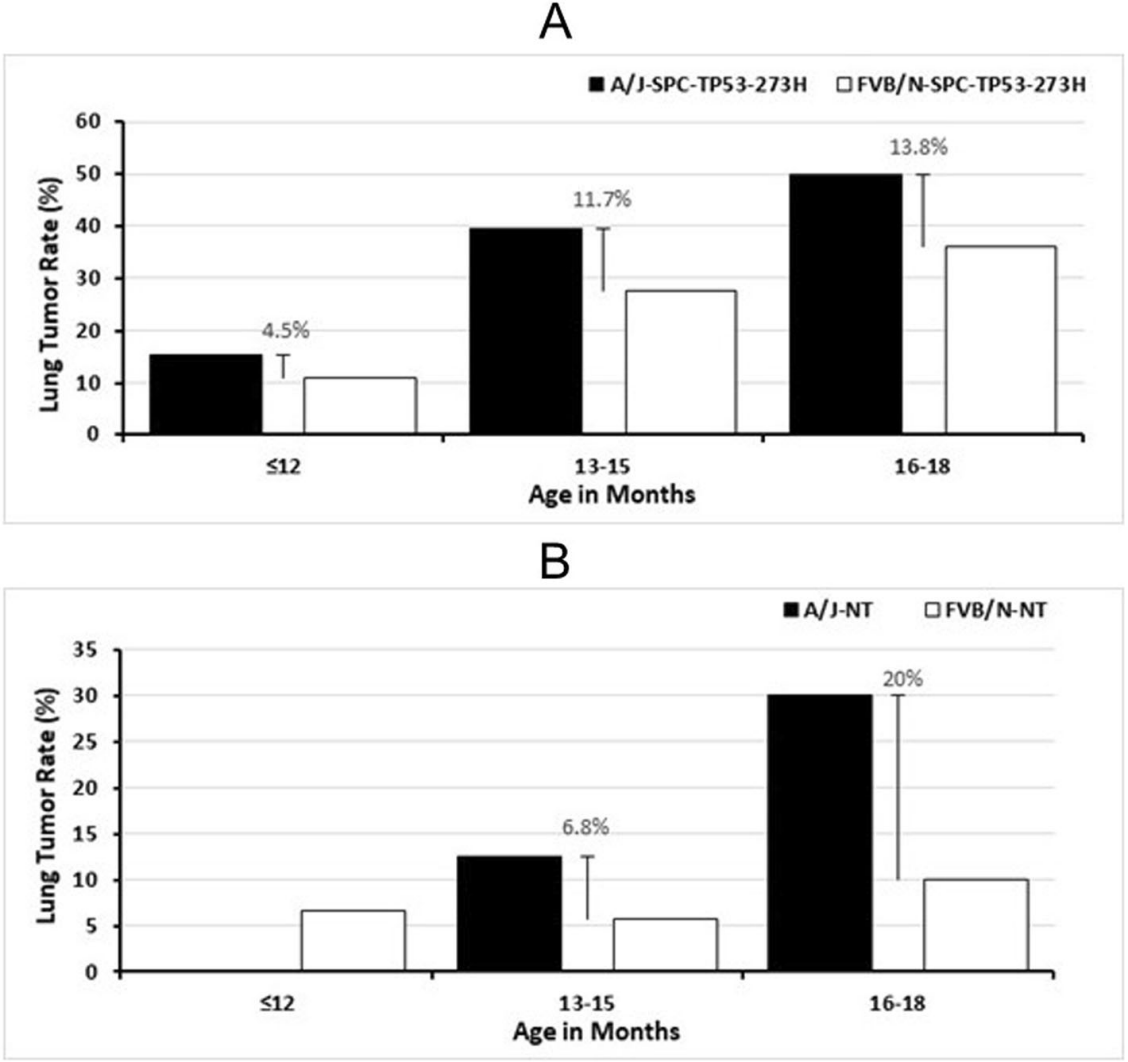
Lung Tumor Rate in FVB/N-SPC-TP53-273H and A/J-SPC-TP53-273H mice. **a** FVB/N-SPC-TP53-273H and A/J-SPC-TP53-273H mice were sacrificed and analyzed for lung tumor incidence. The rate of tumor formation is shown here in both strains. The A/J-SPC-TP53-273H transgenic mice have an increased rate of lung tumor in all age groups when compared to the FVB/N-SPC-TP53-273H mice. The tumor rate change between both groups is represented by the error bar. **b** Lung tumor rate observed in A/J non-transgenic (A/J-NT) mice and FVB/N non-transgenic (FVB/N-NT) mice. At age of 13–15 and 16–18 months A/J- NT mice had a more frequent tumor rate when compared to FVB/N-NT mice

To investigate the lung cancer development in different murine stains, we analyzed tumor prevalence and compared tumor rate between the FVB/N-SPC-TP53-273H and A/J-SPC-TP53-273H mice. Among the 74 A/J-SPC-TP53-273H mice and 148 FVB/N-SPC-TP53-273H transgenic mice investigated (Table 2), we found that A/J-SPC-TP53-273H transgenic mice had a higher lung tumor rate when compared to FVB/N transgenic mice at different age cohorts (Fig. 3a). Overall lung tumor rate difference between A/J transgenic and FVB/N transgenic in all age groups was 11.5%. In addition to the above, we also compared lung tumor formation rate between the A/J non-transgenic (A/J-NT) wildtype mice to FVB/N non-transgenic (FVB/N-NT) wildtype mice. A/J-NT mice had a much higher lung tumor rate when compared to the lung tumor rate in FVB/N-NT mice after 12 months (Fig. 3b). In the 16–18 months cohort, the lung tumor rate tripled in A/J-NT wildtype mice to 30%, whereas FVB/N-NT had a 10% lung tumor rate in the same age cohort.

**Table 2:** Lung tumor formation in FVB/N strain of mice

Tumor	Month ≤ 12	Month 13–15	Month 16–18	***Total***
**FVB/N-SPC-TP53-273H**				
Yes	3	13	17	33
No	51	34	30	115
**Number of mice**	54	47	47	148
**Tumor rate**	**0.06**	**0.28**	**0.36**	**0.22**
**FVB/N-NT**				
Yes	2	2	2	6
No	49	33	18	100
**Number of mice**	51	35	20	106
**Tumor rate**	**0.04**	**0.06**	**0.10**	**0.06**

### Oncogenic potential of mutant TP53-273H in spontaneous lung adenocarcinoma development

To investigate the oncogenic potential of the mutant TP53-273H in different stain of mice, we analyzed the impact of mutant TP53-273H on spontaneous lung cancer development in each strain. We simply compared the lung tumor rate between the transgenic cohort and non-transgenic cohort within a strain and age group. The difference observed in lung tumor rate of the mutant TP53-273H is thereby named oncogenic potential. In the A/J mice strain, the oncogenic potential was 0.095 (9.5%) in the less than 12 months cohort, 0.26 (26%) in the 13–15 months cohort, and 0.20 (20%) in the 16–18 months cohort. Our observed FVB/N mice strain oncogenic potential in different age cohorts was 0.016 (1.6%) in ≤12 cohort, 0.22 (22%) between 13 and 15 months, 0.26 (26%) between 16 and 18 months, and lastly no oncogenic impact was observed between 10 and 12 month (Table 3). Overall a constant oncogenic potential of the mutant TP53-273H was found in both A/J and FVB/N strains (t = 0.74).

**Table 3:** Oncogenic potential of mutant TP53-273H in A/J and FVB/N mice

	Month ≤ 12	Month 13–15	Month 16–18	Overall
**A/J-SPC-TP53-273H tumor rate**	0.095	0.394	0.500	0.338
**A/J-NT tumor rate**	0.000	0.136	0.300	0.143
**Oncogenic potential in A/J**	**0.095**	**0.258**	**0.200**	**0.184**
**FVB/N-SPC-TP53-273H tumor rate**	0.056	0.277	0.362	0.223
**FVB/N-NT tumor rate**	0.039	0.057	0.100	0.057
**Oncogenic potential in FVB/N**	**0.016**	**0.219**	**0.262**	**0.166**

### Lung tumor growth rate in the FVB/N-SPC-TP53-273H mice

**Fig. 4:**
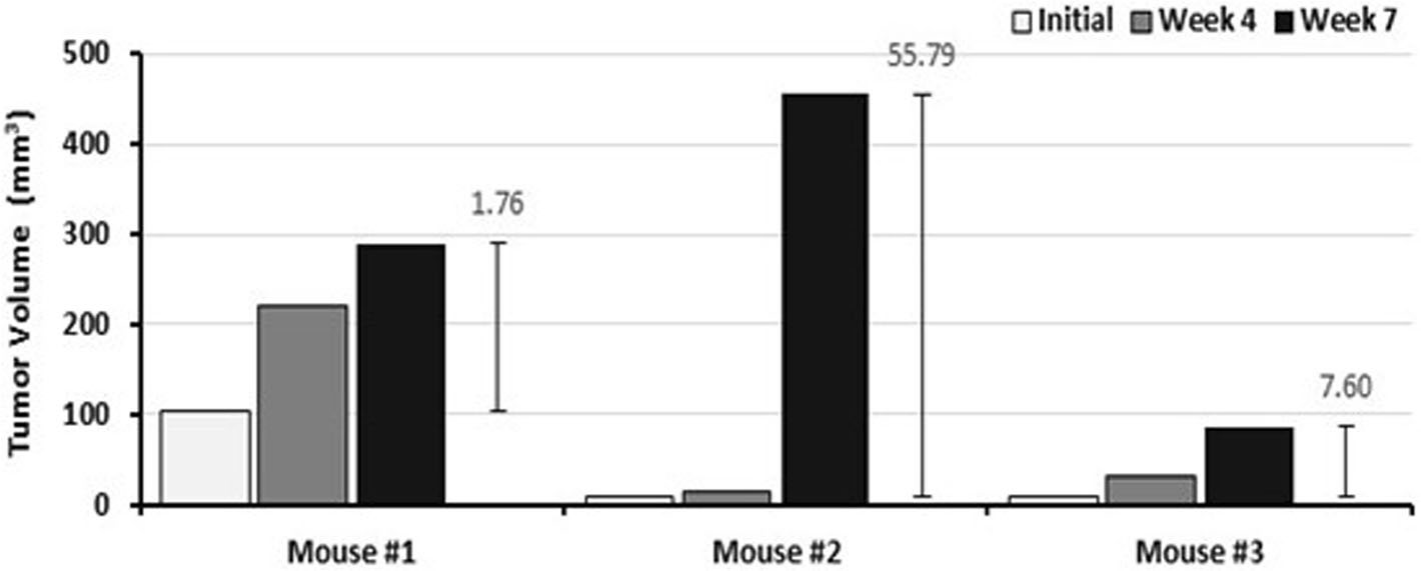
Spontaneous lung tumor development in three FVB/N-SPC-TP53-273H mice over 7 weeks. Three mice were followed over a total time of 7 weeks and analyzed for lung tumor growth at week 1 (initial), week 3 and week 7 via micro CT imaging. The tumor volume progress was recorded from initial to week 7 in three selected mice. The fold change in tumor volume between initial and week 7 is depicted by the error bars for mice 1, 2 and 3

One of the most important characteristics for a successful in vivo cancer model suitable for treatment is that the model should provide a sufficient window for the therapy application and evaluation of new treatment approaches. For this reason, we investigated lung tumor progression patterns in FVB/N-SPC-TP53-273H mice with a micro CT. At the age of 12 months, a selection of FVB/N-SPC-TP53-273H mice were screened for lung tumor formation with a micro CT. Three tumor bearing mice were evaluated for tumor growth. After the initial screening, the lung tumors were followed up every 3 weeks starting with week 1, for a total of 7 weeks, again using micro CT imaging. These images showed that tumor volume increases in all three mice over time, reaching a peak at week 7 (Fig. 4).

**Fig. 5:**
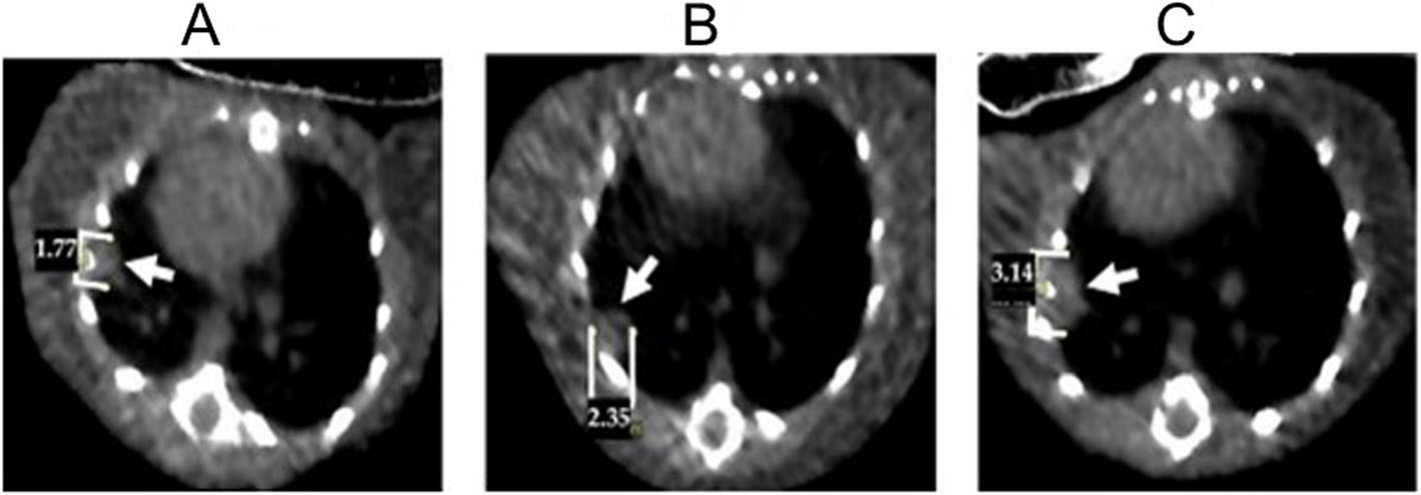
Lung tumor growth over time assessed by micro CT. Micro CT images were taken from a FVB/N-SPC-TP53-273H transgenic mouse and followed over a period of 7 weeks. **a** Initial scan of a mouse lung showing a single tumor with diameter of 1.77 mm. **b** Lung tumor increasing in size to about 2.35 mm in diameter at week 4. **c** At week 7 tumor size was observed to be 3.14 mm

Although a massive tumor volume increase was observed in one mouse after monitoring week 4 (mouse #2 in Fig. 4), all mice survived more than 8 weeks after initial lung cancer diagnosis via micro CT. Figure 5 shows that tumor size increases over time in a FVB/N-SPC-TP53-273H transgenic mouse. Initial scans show development of a single tumor with a diameter size of 1.77 mm, which increased over time to 3.14 mm.

## Discussion

During the past two decades scientists have developed a variety of lung cancer treatments which have proved to be efficient in combating disease manifestations and enabling further research on gene alterations and their effect on lung cancer development [38]. One option is the epidermal growth factor receptor (EGFR) tyrosine kinase inhibitor. When the tyrosine kinase receptor EGFR experiences a spontaneous mutation, the mutant EGFR protein leads to uncontrolled cell proliferation [39]. Studies on EGFR mutation has led to the development and approval of several drugs by the FDA which block EGFR receptor specifically such as erlotinib and gefitinib [40]. Another molecular target in treating lung cancer is the anaplastic lymphoma kinase (ALK) fusion gene [41]. ALK rearrangements occurring in the ALK kinase domain along with EML4, NPM and TFG have been identified to exhibit oncogenic activity by hyperactivating ALK, thus creating inversions or translocations on chromosome 2 that fuse variable regions of a partner gene with exon 20 of the ALK gene [42–44]. This discovery has led to an increased understanding of ALK’s role in disease metastasis, and subsequently, the development of targeted drugs. The incidence of tumor associated EGFR mutation and anaplastic lymphoma kinase (ALK) rearrangement varies from 10% (in the USA) to 35% (in East Asia) and 5–7%, respectively, in patients with NSCLC [45–48]. The use of tyrosine kinase inhibitors targeting EGFR and ALK subpopulations have resulted in significant patterns of clinical practice [49–51].

In the last few years, treatment of patients with non-small cell lung cancer (NSCLC) has impressively benefitted from immunotherapy, in particular from the inhibition of immune checkpoints such as programmed cell death-1 (PD-1) and its corresponding cell death ligand-1 (PD-L1) [52–57]. Subsequently, immune checkpoint inhibitors (ICI) on T-cell stimulation facilitate immune mediated elimination of tumor cells [58]. These antibody mediated therapies have then shown to produce beneficial effects against many malignancies and now play a major role in advanced lung cancer management [42]. Early clinical trials with drugs such as nivolumab, pembrolizumab or avelumab have shown rapid and durable responses in about 14–20% of pre-treated patients with advanced NSCLC [59–66]. However, concrete evidence suggests that only a small portion of lung cancer patients benefit from this treatment and some patients showed severe immune-related adverse events and systemic autoimmune responses [67]. Unfortunately, very little is known regarding the mechanisms underlying acquired resistance to immune checkpoint inhibitor therapy [56]. It is clear that additional studies are needed to explore the mechanisms behind the resistance to both immune checkpoint inhibitor therapy and targeted therapies, as well as to develop robust pre-clinical in vivo models to evaluate novel treatments with better prediction of their effects in humans.

Our spontaneous non-small cell lung cancer models reported here would provide a valuable tool for evaluating personalized therapeutic strategies. Different from other lung tumor animal models, our lung tumor model limits the damage to the lung. Further, mutations in the KRAS gene, which are acquired mutations, closely mimic the events that lead to spontaneous lung cancer development in humans. More importantly, our model is a “treatable” model, as these mice develop a single lung tumor that is easy to follow up, in contrast to other engineered lung tumor models (e.g. KRAS) which develop multiple lung tumors. In addition, our lung cancer model serves as a treatable model because these tumor bearing mice survive more than 8 weeks after initial detection of lung cancer with a micro CT. Thus, our models provide a sufficient window for evaluating new treatment strategies.

**Fig. 6:**
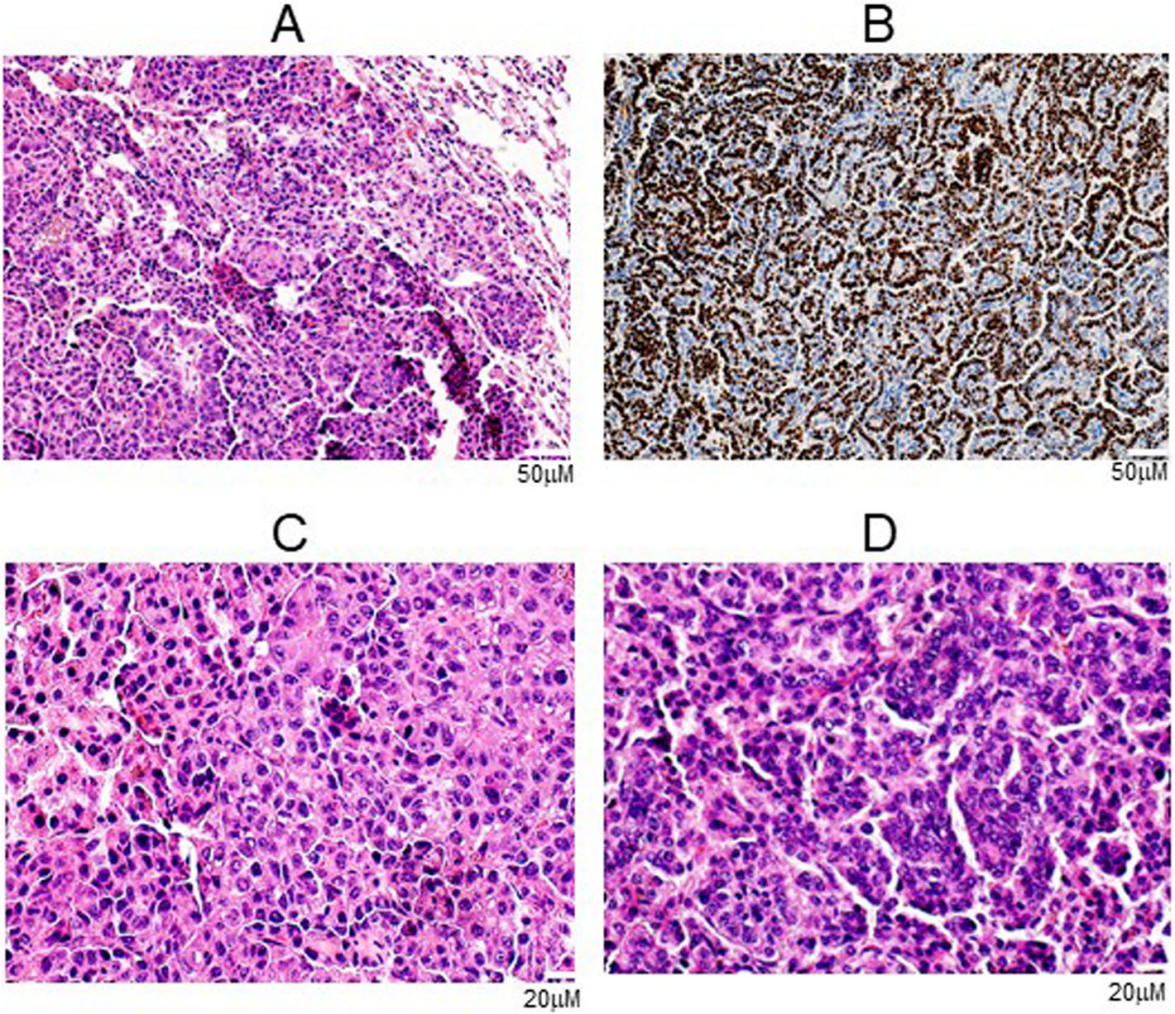
Histopathology of lung cancer in two lines of transgenic mice. **a** H&E staining of an invasive adenocarcinoma from an A/J-SPC-TP53-273H mouse, **b** immunohistochemical detection of human mutant p53-273H expression in a murine lung adenocarcinoma collected from an A/J-SPC-TP53-273H mouse. The antibody used was an anti-human specific p53 antibody (DO-7), **c** H&E staining of an adenocarcinoma from an A/J-SPC-TP53-273H mouse at high magnification (400X), and **d** H&E staining of an adenocarcinoma from an FVB/N-SPC-TP53-273H mouse at high magnification (400X)

From a histopathological perspective the lung tumors in our mice model resemble human adenocarcinoma, a major type of non-small cell lung cancer in humans. Both lines of transgenic mice developed lung adenocarcinomas and human mutant p53 protein was expressed in the tumors (Fig. 6). These lung tumors exhibited areas of variant histology, including areas of clear secretory change, areas of high nuclear grade, areas of oncolytic change and areas of solid proliferation. These variant histological patterns are evidence of dedifferentiation, a phenomenon which human lung tumors readily exhibit [36].

Our results further demonstrated that the human mutant TP53-273H has a similar oncogenic potential that essentially initiates lung cancer formation in both FVB/N and A/J stains. First, we found that a single spontaneous lung adenocarcinoma developed in both FVB/N and A/J mice. After comparing lung tumor rates between the FVB/N-NT (wild type) mice and the A/J-NT (wild type) mice, we deduced that overall the wild type FVB/N-NT mice are less sensitive to develop a spontaneous tumor (Fig. 3b, Tables 1 and 2). Furthermore, by comparing lung tumor rates between FVB/N-SPC-TP53-273H and A/J-SPC-TP53-273H transgenic mice, we found that the A/J-SPC-TP53-273H transgenic mice have a higher lung cancer rate (Fig. 3a), which may be due to an increased sensitivity in this strain. However, when we compared the oncogenic potential (tumor rate difference between the transgenic mice and non-transgenic mice within a strain and age range), we found the lung tumor rates caused by the human mutant TP53-273H gene were similar between FVB/N and A/J mice (Table 3). For example, at an age of 13–15 months the oncogenic potential of the mutant TP53-273H gene in A/J strain was 0.26 (26%). In the same age range, the oncogenic potential of the mutant TP53-273H gene in FVB/N strain was 0.22 (22%). This indicates that the oncogenic potential observed due to the mutant TP53-273H gene is unique regardless of the fact that A/J mice exhibit higher susceptibility to spontaneous and chemically induced lung cancer [68, 69]. Since FVB/N mice are larger in litter size when directly compared to A/J stain, we think the FVB/N transgenic mice would provide a better platform for anti-cancer treatment evaluations. As the mutant TP53 gene is under the control of the surfactant protein C promoter, these mice develop tumors only in lung tissue. Additionally, these mice have sufficient immune components that resemble the human immune system and deliver a good platform for evaluating immune checkpoint inhibitors in treatment of spontaneous lung cancer.

On the other hand, the A/J inbred strain is widely used in cancer and immunology research. Chemical induction of lung tumors in A/J mice has been demonstrated from the early 1940s [70, 71]. This mouse line has been used extensively to identify both environmental carcinogens and chemo-preventive agents for lung cancer [68]. The histological, morphological, biochemical, growth, and transplantation characteristics of lung tumors induced in A/J mice have been well documented [72]. It is well known that the mutation load is increased in at-risk individuals including the elderly, smokers, and people carrying germline mutations. Therefore, the A/J-SPC-TP53-273H mice could be a valuable line for studying the interaction between p53 mutation and environmental carcinogens, like cigarette smoke.

## Conclusion

The mutant TP53-273H is one of the most common genetic mutations in human lung cancer. A consistent oncogenic potential was observed when the mutant TP53-273H gene was expressed in A/J and FVB/N strains. While many in vivo models are not currently being suitable for evaluating immune checkpoint inhibitors, we believe that our model is an ideal candidate for testing novel immunotherapeutic options in treatment of lung cancer.

## Data Availability

The construct used for creating the transgenic mouse is available from the corresponding author.

## Abbreviations

NSCLC: Non-small cell lung cancers
TP53: Tumor protein p53
ALK: Anaplastic lymphoma kinase
SPC: Surfactant protein C
EGFR: Epidermal growth factor receptor
KRAS: Kirsten Rat Sarcoma Viral Proto-Oncogene
ULAR: University Laboratory Animal Resources
PD-1: Programmed cell death-1
PDL-1: Programmed cell death ligand-1
ICI: Immune checkpoint inhibitors
